# Influence of Resistance Training Proximity-to-Failure, Determined by Repetitions-in-Reserve, on Neuromuscular Fatigue in Resistance-Trained Males and Females

**DOI:** 10.1186/s40798-023-00554-y

**Published:** 2023-02-08

**Authors:** Martin C. Refalo, Eric R. Helms, D. Lee Hamilton, Jackson J. Fyfe

**Affiliations:** 1 Centre for Sport Research (CSR), School of Exercise and Nutrition Sciences, Deakin University, Geelong, Australia; 2 Sport Performance Research Institute New Zealand (SPRINZ), Auckland University of Technology, Auckland, New Zealand; 3 Institute for Physical Activity and Nutrition (IPAN), School of Exercise and Nutrition Sciences, Deakin University, Geelong, Australia

**Keywords:** Resistance training, Fatigue, Proximity-to-failure, Repetitions-in-reserve

## Abstract

**Background:**

This study examined the influence of proximity-to-failure in resistance training (RT), using subjective repetitions-in-reserve (RIR) prediction, on neuromuscular fatigue and perceptual responses.

**Methods:**

Twenty-four resistance-trained males (*n* = 12) and females (*n* = 12) completed three experimental trials in a randomised order, each involving six RT sets (barbell bench press) with 75% 1-RM performed to either momentary muscular failure (FAIL), 1-RIR, or 3-RIR. Changes in lifting velocity with a fixed load were assessed from pre-exercise to post-exercise with the aim of quantifying *acute* neuromuscular fatigue (4 min post-exercise) and the associated time course of recovery (24 and 48 h post-exercise), and from the first to final set performed. Perceptual responses to RT were assessed at multiple time points during and following RT.

**Results:**

Decreases in lifting velocity at 4 min post-exercise were greater for FAIL ( − 25%) versus 1-RIR ( − 13%) and 3-RIR ( − 8%), with greater decreases for male ( − 29%) versus female ( − 21%) participants following FAIL. At 24 h post-exercise, decreases in lifting velocity were greater for FAIL ( − 3%) and 1-RIR ( − 3%) versus 3-RIR (+ 2%), with all between-protocol differences diminishing at 48 h post-exercise. Loss of lifting velocity from the first to final set was greater for FAIL ( − 22%) versus 1-RIR ( − 9%) and 3-RIR ( − 6%), with a greater lifting velocity loss from the first to final set for males ( − 15%) versus females ( − 9%). As proximity-to-failure neared, ratings of perceived discomfort, exertion, and muscle soreness increased, general feelings worsened, and perceived recovery decreased.

**Conclusion:**

These findings support a *linear* relationship between RT proximity-to-failure and both *acute* neuromuscular fatigue and negative perceptual responses, which may influence long-term physiological adaptations and adherence to RT.

**Supplementary Information:**

The online version contains supplementary material available at 10.1186/s40798-023-00554-y.

## Key Points

- In resistance-trained individuals, we observed that *acute* neuromuscular fatigue increased as proximity-to-failure neared (FAIL > 1-RIR > 3-RIR) and was greater for males versus females when RT was performed to momentary muscular failure (FAIL).
- A slight decrement in neuromuscular function when RT was performed to momentary muscular failure and 1-RIR was sustained at 24 h post-exercise versus 3-RIR, with 48 h of recovery post-exercise likely sufficient for complete recovery of neuromuscular function when RT is performed for six sets on the barbell bench press, independent of the proximity-to-failure reached.
- Proximity-to-failure seems to be a key determinant of the perceptual responses to RT, evidenced by the general trend observed for perceptual responses to be more negative as proximity-to-failure neared.
- To our knowledge, these findings are the first to provide evidence for a *linear relationship* between proximity-to-failure (determined by subjective RIR prediction) and both *acute* neuromuscular fatigue and negative perceptual responses to RT.

## Introduction

Proximity-to-failure is defined as the number of repetitions remaining in a resistance training (RT) set prior to momentary muscular failure (i.e. when the concentric portion of a given repetition cannot be completed with a full range-of-motion without deviation from the prescribed exercise form) [1]. As proximity-to-failure nears in a given set, type II skeletal muscle fibres are required to produce higher forces [2, 3], ultimately exposing the active musculature to greater mechanical tension and influencing the subsequent physiological adaptation(s) induced. Neuromuscular fatigue consequent to RT also increases as proximity-to-failure nears [4], potentially impairing contractile function during and subsequent to RT and ultimately hampering maximal strength development or muscle hypertrophy by reducing the absolute load lifted or the exposure of muscle fibres to mechanical tension [5], respectively. This understanding highlights the importance of investigating the specific effect of different proximities-to-failure on neuromuscular fatigue, along with the associated time courses of recovery, which are practically important for RT prescription to maximise long-term physiological adaptations.

A key barrier to understanding the influence of proximity-to-failure on neuromuscular fatigue and other short-term responses (e.g. muscle damage, perceived discomfort and exertion, general feelings, perceived recovery, etc.) that may negatively influence physiological adaptations to RT is the current set termination prescriptions used in research investigating proximity-to-failure [1]. Firstly, no consensus definition of ‘failure’ exists in the literature, and as such, studies employ various definitions of *set failure* (i.e. umbrella term describing the set termination criteria applied to ‘failure’ in a given study) that alter the RT stimulus achieved and do not provide an accurate insight into the true effect of reaching momentary muscular failure during RT. Although momentary muscular failure is the most objective definition of set failure, our recent scoping review [1] only identified six studies (out of 25) that assessed the influence of proximity-to-failure on short-term responses to RT and explicitly stated that the definition of momentary muscular failure was employed. Further, a recent meta-analysis found greater increases in neuromuscular fatigue and muscle damage after RT performed to set failure versus non-failure [6]; however, these findings are limited to males and considering the potential for biological sex differences in neuromuscular fatigability [7], how proximity-to-failure influences short-term responses to RT in females requires future investigation. It is also likely that the proximity-to-failure reached by participants in non-failure conditions varies considerably within- and between-studies due to commonly employed predetermined repetition prescriptions and individual variability in the maximum number of repetitions possible with a given load [8–10]. Some studies have attempted to address this research limitation by employing ‘velocity loss’ thresholds to control and standardise set termination; however, even the magnitude of velocity loss achieved during a given set cannot accurately inform proximity-to-failure during RT [1] as evidenced by one study that found participants who performed the squat exercise until 40% velocity loss reached momentary muscular failure ~ 56% of the time [11]. As such, although mechanical and metabolic indicators of neuromuscular fatigue increase with the magnitude of velocity loss achieved [12–14], the proximity-to-failure reached across velocity loss conditions is unknown and likely varies. Taken as a whole, neuromuscular fatigue is greater when RT is performed to set failure versus non-failure and increases as the magnitude of velocity loss rises (and theoretically, as proximity-to-failure nears), but inconsistencies in the literature regarding the proximity-to-failure achieved during RT limit understanding of the influence of proximity-to-failure on neuromuscular fatigue and other short-term responses to RT.

Quantifying the proximity-to-failure reached during RT with the number of repetitions-in-reserve (RIR) is emerging as a popular strategy that requires set termination to occur once the individual performing RT believes they can only perform a certain number of full repetitions before reaching momentary muscular failure. This ‘subjective RIR prediction’ was recently tested in a study comparing the effect of RT performed to 3-RIR versus momentary muscular failure on neuromuscular fatigue. While neuromuscular fatigue was similar between conditions [15], limitations with the nature of instruction provided to participants meant set termination may have varied between 0 and 3-RIR in the 3-RIR condition, limiting insight into the specific effect of proximity-to-failure on neuromuscular fatigue. Few studies have investigated the effect of subjective RIR prediction on RT outcomes [15–18], likely due to the many factors that may influence the accuracy of subjective RIR predictions (e.g. accuracy is improved when RIR prediction is performed closer to momentary muscular failure [19], as the relative load lifted and the number of successive sets performed increases [20, 21], and in resistance-trained versus untrained individuals [22, 23]). Nonetheless, subjective RIR prediction is likely the most practical method of controlling proximity-to-failure during RT as it can be easily implemented in an RT prescription (e.g. 3 sets of 10–15 repetitions with 2-RIR), particularly in resistance-trained individuals, and its rigorous application in research may address current methodological limitations and help better translate findings to practical recommendations.

### Objectives

The primary objective of this study was to examine the influence of RT proximity-to-failure on the level of neuromuscular fatigue incurred in resistance-trained males and females. Therefore, we assessed changes in lifting velocity (i.e. mean velocity of the concentric portion of a repetition), a valid indicator of neuromuscular fatigue [24], with a fixed load from (i) pre-exercise to post-exercise with the aim of quantifying *acute* neuromuscular fatigue (4 min post-exercise) and the associated time course of recovery of neuromuscular function (24 and 48 h post-exercise), and (ii) from the first to the final set performed. We also assessed biological sex differences in *acute* neuromuscular fatigue. Perceptual responses to RT were also assessed, including perceptions of discomfort, recovery, exertion, muscle soreness, and general feelings. We hypothesised that reaching closer proximities-to-failure during RT would induce greater neuromuscular fatigue at all post-exercise time points and greater subjective perceptions of discomfort, exertion, muscle soreness, and reduced recovery. Further, we expected neuromuscular fatigue to be lower in females compared to males.

## Methods

### Experimental Approach

**Fig. 1:**
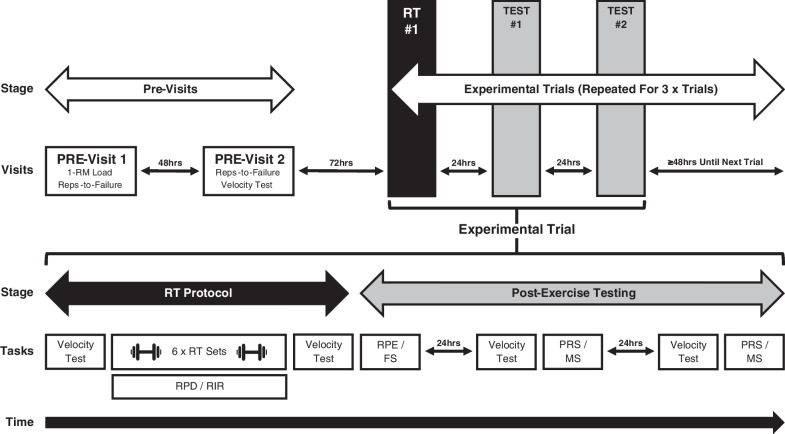
Schematic overview of study design and experimental trials. Participants completed two pre-visit sessions and three experimental trials. Each trial consisted of a resistance training protocol (FAIL, 1-RIR, or 3-RIR) involving six sets performed on the barbell bench press exercise (75% 1-RM load), whereby changes in lifting velocity were assessed from the first to the final set and from pre-exercise to 4 min post-exercise. RPD was assessed after the completion of each set, and RPE and FS were assessed after the completion of the RT protocol. Following the RT protocol, two testing sessions 24 and 48 h thereafter were also completed to assess the recovery time course of lifting velocity, MS, and PRS. FS, feeling scale; MS, muscle soreness; PRS, perceived recovery status; RIR, repetitions-in-reserve; RPD, rating of perceived discomfort; RPE, rating of perceived exertion; RT, resistance training

This was a randomised crossover trial (conducted at JPS Health & Fitness, Melbourne) whereby participants attended two pre-visit sessions and three experimental trials, each trial involving one resistance training session followed by two testing sessions (24 and 48 h post-exercise) (Fig. 1). In pre-visit one, the 1-RM load was determined for the flat barbell bench press (BP) exercise and used to inform load selection during each experimental RT protocol (75% 1-RM). A repetitions-to-failure assessment (Sect. ‘Pre-Visit Sessions’) was also conducted for the BP in pre-visit one and two. After the pre-visits, participants completed three experimental trials that involved RT protocols performed to either momentary muscular failure (defined as: the point where despite attempting to do so, the individual was unable to complete the concentric portion of their current repetition with a full range-of-motion without deviation from the prescribed form of the exercise) or to a subjectively predicted 1-RIR or 3-RIR. To provide surrogate measures of neuromuscular fatigue consequent to RT, changes in lifting velocity were assessed from the first to the final set, and from pre-exercise to post-exercise (4 min, 24 h, and 48 h post-exercise). Perceived muscle soreness and recovery were also assessed 24 and 48 h post-exercise. To assess perceptual responses to RT, participants rated their perceived discomfort after the completion of each set, and their general feelings and perceived exertion upon completion of each RT protocol.

### Subjects

Pre-exercise participant characteristics are presented in Table 1. A total of 12 males and 12 females were recruited. All participants: (i) were between 18 and 40 years old, (ii) had no existing musculoskeletal injuries or neuromuscular disorders, (iii) confirmed they had not used anabolic steroids or any illegal agents known to increase muscle size for the previous year, and (iv) had a minimum of 3 years of RT experience involving a minimum of three or more RT sessions completed per week. The mean 1-RM for the bench press exercise was also greater than 120% and 80% of bodyweight for males and females, respectively, indicating an advanced sample of participants as specified by Santos Junior et al. [25]. All participants reported experience working with a private fitness coach in a face-to-face setting, 12 participants declared they had previously competed in strength or physique sports (e.g. powerlifting or bodybuilding), and 23 participants reported experience with subjective RIR prediction.

**Table 1:** Baseline participant characteristics

Variable	Men (*n* = 12)		Females (*n* = 12)	
	Mean ± SD	Range	Mean ± SD	Range
Age (y)	28.50 ± 5.3	19–39	31.58 ± 5.70	23–40
Bodyweight (kg)	85.1 ± 8.3	74–99	62.3 ± 11.0	52–90
RT experience (y)	8.3 ± 3.7	3–15	7.2 ± 2.3	4–13
RT frequency (per wk)	4.4 ± 0.7	3–5	4.4 ± 0.7	4–6
1-RM BP (kg)	116.0 ± 20.8	92.5–157.5	54.9 ± 13.0	35–77.5
Relative strength	1.37 ± 0.26	1.14–1.93	0.88 ± 0.17	0.56–1.08

An overview of the relevant characteristics for each participant. Relative strength calculated as: barbell bench press 1-RM (kg) divided by bodyweight (kg).

1-RM, one repetition maximum; BP, bench press; kg, kilograms; per wk, per week; RT, resistance training; y, years

#### Sample Size Justification

The target sample size of 24 participants was based on the following pragmatic considerations: (i) recruiting more than 24 participants was not feasible given resource constraints including the time and costs associated with data collection and subsequent analyses, and (ii) the chosen sample size is greater than most published studies investigating the influence of RT proximity-to-failure on neuromuscular fatigue using similar research designs [4, 13, 26–28]. An α-priori sample size calculation was therefore not performed for this study. Instead, a sensitivity power analysis was performed in G*Power software (Version 3.1.9.7) using an ANOVA: repeated-measures, within-between interaction to determine the minimum (critical) effect size (Cohen’s *d* = 0.27) for between-protocol differences in loss of lifting velocity (from the first to final set for a given exercise) that could be statistically rejected based on a pre-specified sample size (*n* = 24) and both type I (0.05) and type II (0.20) error rates. Given previous research [24] reported an effect size of *d* = 2.5 for the difference in velocity loss between RT performed to set failure versus non-failure (i.e. a 12-RM versus 10 repetitions with the 12-RM load), we considered a critical effect size of *d* = 0.27 sufficient to detect/reject likely effect sizes for between-protocol differences in this study.

### Procedures

#### Exercise and Nutrition Control

Participants were asked to not perform any RT or high-intensity aerobic exercise in the 24 h period before each study visit to minimise any potential confounding influences on outcome measures. To ensure recovery and performance were not influenced by sub-optimal nutritional status, participants consumed sufficient protein (2 g/kg body mass) and energy based on their body weight and estimated energy expenditure (at minimum, energy intake was matched with total daily energy expenditure) consistent with published guidelines [29]. Considering the number of study visits required, it was not feasible for participants to replicate their nutritional intake before each study visit. As such, participants were asked to track their nutritional intake on a food tracking application and measure their bodyweight each week to ensure that no weight loss occurred.

#### Menstrual Cycle Considerations

Upon recruitment, female participants started using a menstruation diary to ensure accurate information regarding the menstrual cycle was retrieved and recorded for future use. When possible (based on scheduling and practical constraints), females commenced their experiential trials in the early follicular phase of their menstrual cycle where the ratio between oestrogen and progesterone is small [30]. For this reason, oral contraceptive use was not controlled for, as endogenous oestrogen and progesterone levels are similar in the early follicular phase for females that are eumenorrheic and using oral contraceptives [31]. Participants that were amenorrheic (*n* = 2) were permitted to start their experimental trials at any time. If participants experienced menstrual symptoms during the study period that were perceived to affect training performance, study visits were rescheduled as necessary. Notably, recent meta-analyses indicate that both (i) the current menstrual cycle phase [32] and (ii) modern oral contraceptive use [33] have at most trivial effects on exercise performance at the *group* level.

#### Pre-visit Sessions

Approximately 1 month before the commencement of the study period (depending on participant availability and time constraints), participants underwent a pre-study familiarisation to establish appropriate exercise technique with maximal intended lifting velocity. Participants performed two sets of five repetitions with the minimum load on the BP exercise to ensure appropriate technique as follows: the advanced participants employed and replicated their own lifting grip based on their previous experience with the BP exercise (at minimum, the barbell had to be grasped slightly outside shoulder width) and lowered the barbell until it contacted their chest (below the nipple line) and then lifted it back to the starting position without excessive bouncing off the chest, or raising of the shoulders, trunk, or glutes off the bench. Participants were instructed to perform the concentric (lifting) phase of each repetition with maximal lifting velocity (i.e. as fast as possible), followed by a controlled eccentric (lowering) phase (~ 2 s). The amount of time in-between repetitions (maximum of one breath) was kept consistent throughout the whole set. Similar to previous research [34], the mean concentric velocity (i.e. described herein as the ‘lifting velocity’) for each repetition was measured using a linear position transducer (GymAware, Kinetic Performance Technology, Canberra, Australia) attached to the one side of the barbell (just inside the collar). If fluctuations in the lifting velocity were identified across successive repetitions, the participant was required to attempt another set of five repetitions until a similar lifting velocity was achieved on each repetition (i.e. a range of ≤ 0.02 m/s across repetitions). Once the lifting velocity achieved was within ≤ 0.02 m/s across repetitions, an additional load (15–20 kg for males and 5–10 kg for females) was added, and participants performed another set of three repetitions with maximal intended lifting velocity. Once participants were familiarised with this lifting strategy, they were told to incorporate the BP into their own RT regimen and continue practising with maximal intended lifting velocity until the commencement of the study.

In pre-visit one, after re-familiarisation with the correct exercise technique, participants completed a 1-RM assessment for the BP. First, a warm-up consisting of one set of five repetitions was performed with the minimum possible load (20 kg). The load was then progressively increased (15–20 kg increments for males and 5–10 kg for females) until the lifting velocity was lower than 0.5 m s^−1^. Thereafter, the load was increased in smaller increments (2.5–10 kg for males and 1.25–5 kg for females) until the 1-RM was determined, defined as the heaviest load with which a single repetition was possible with a full range-of-motion. For the lighter loads (> 1 m s^−1^), three repetitions were performed at each load, two repetitions were performed for the moderate loads, and a single repetition for the heavier loads (< 0.5 m s^−1^). Three minutes of passive recovery was allowed between sets for lighter and moderate loads, and approximately five minutes of passive recovery for heavier loads. If the participant was unable to complete a repetition at a given load, they were allowed one additional attempt at that load. If the second attempt was not successful or if the participant declined a second attempt, the load was either (i) reduced to 50% of the difference between it and the last successful 1-RM attempt, or (ii) the last successful repetition was confirmed as the 1-RM.

Once the 1-RM assessment was complete, and in pre-visit two after a standardised warm-up, participants were required to complete a repetitions-to-failure assessment that involved performing two sets to momentary muscular failure with the load corresponding to 75% of 1-RM. Participants were first briefed about subjective RIR prediction, and it was made clear that 0-RIR indicates the last full range-of-motion repetition possible before momentary muscular failure is reached (i.e. if a subsequent repetition was attempted, momentary muscular failure would occur). Before each set to momentary muscular failure, participants were given an RIR target (1- or 3-RIR in a randomised order) and were required to verbally indicate when they believed they had reached the RIR target during the set. Considering the possibility for participants to conflate subjective RIR predictions with perceptions of discomfort, participants were also briefed about the difference between perceived discomfort and subjective perception of proximity-to-failure. After verbal indication, participants were required to continue performing repetitions until momentary muscular failure occurred to assess the accuracy of RIR prediction. The additional repetitions performed after the participant provided the verbal indication were counted to assess individual predictive ability and were recorded for future analysis. At no point were participants informed about the number of repetitions completed within a set, nor were the repetitions counted aloud throughout the set by the supervisors. Participants also rated their level of perceived discomfort after completing each set using the rating of perceived discomfort (RPD) scale (Sect. ‘Perceived Discomfort’). During pre-visit two, a velocity assessment (Sect. ‘Assessment of Recovery Time-Course’) was also conducted for familiarisation purposes, and upon completion of the familiarisation session participants were asked to rate their perceived exertion and general feelings associated with the RT performed.

#### Experimental Trials

The RT protocols (Fig. 1) completed during each experimental trial consisted of the BP exercise performed with 75% 1-RM. Three experimental trials were conducted, involving RT protocols performed in a randomised order: (i) momentary muscular failure (FAIL), (ii) 1-RIR, and (iii) 3-RIR. A minimum of 96 h was allocated between each RT protocol to ensure adequate recovery and minimise the influence of residual fatigue on subsequent trials. Before the commencement of each RT protocol, four warm-up sets were performed, starting with the minimum load for each exercise and working up to 50%, 65%, and 85% of the 75% 1-RM load (for six, five, four and three repetitions, with 2-min inter-set rest periods). A pre-exercise velocity assessment was then completed (Sect. ‘Assessment of Recovery Time-Course’) before six total sets were performed until the target proximity-to-failure of the protocol was reached (repetitions performed differed between participants). Set termination for the RIR protocols involved the participant subjectively terminating each set when they perceived they had reached the RIR target (1- or 3-RIR) with no physical or verbal assistance from the supervisors. Participants were therefore provided with the following standardised instruction: *‘you will be required to stop the set when you perceive to have n (1 or 3, depending on the RIR target of the protocol) repetitions-in-reserve.’* Conversely, during the FAIL protocol, set termination occurred when the supervisor was required to assist the participant in re-racking the barbell due to the participant being: (i) unable to lift the barbell off their chest, despite attempting to do so, (ii) unable to complete a full range-of-motion repetition despite being provided with two seconds to lift the bar beyond the sticking point (i.e. the point during the concentric phase where the barbell stopped moving upwards), or (iii) the barbell started exhibiting downward motion during the concentric phase. Four minutes of passive recovery was allowed between sets, and upon completion of the sixth (and final) set, participants rested for another four minutes and repeated the velocity assessment to establish an immediate measure of acute neuromuscular fatigue. Participants were also required to rate their perceived discomfort after each set (Sect. ‘Perceived Discomfort’), and their perceived exertion and general feelings after completing each RT protocol (Sect. ‘Perceived Exertion and General Feelings’). Participants also attended the training facility 24 and 48 h thereafter to rate their perceived recovery and perceived muscle soreness (Sect. ‘Perceived Recovery and Muscle Soreness’) before completing another velocity assessment to assess the post-exercise recovery time course of neuromuscular function.

#### Objective Outcome Measures

##### Assessment of Recovery Time Course

Three BP repetitions were performed (with maximal intended lifting velocity) using 85% of the 75% 1-RM load before the commencement of each RT protocol (i.e. last warm-up set), and 4 min, 24 h, and 48 h following the completion of each RT protocol (a standardised warm-up was completed 24 and 48 h post-exercise) (Fig. 1). The change in the mean lifting velocity of the three repetitions from pre-exercise to post-exercise was used as a surrogate measure of *acute* neuromuscular fatigue (4 min post-exercise) and the associated recovery time course of neuromuscular function (24 and 48 h post-exercise). Strong verbal encouragement and velocity feedback were provided during each repetition to ensure participants were applying maximal intended lifting velocity.

##### Loss of Lifting Velocity from First to Final Set

The lifting velocity achieved in each set performed (i.e. mean lifting velocity of all repetitions completed within each set) was calculated to determine the decline in mean lifting velocity from the first to the final set and was used as a surrogate measure of the *acute* neuromuscular fatigue incurred over the six sets.

##### Repetition Loss from First to Final Set

To determine the influence of proximity-to-failure on RT volume (considering all participants lifted a relative load equal to 75% 1-RM, RT volume was calculated as: volume = sets * repetitions), the total number of repetitions achieved in each set was recorded to determine the volume accumulated within each RT protocol and the percentage decrease in repetitions performed from the first set to the final set.

#### Subjective Outcome Measures

##### Perceived Discomfort

Immediately after completion of each RT set, participants rated their perceived discomfort using a rating of perceived discomfort (RPD) scale [35]. Participants were asked*: ‘how much discomfort did you feel in that set?’* and to rate their perceived discomfort during the set on a 1–10 scale, whereby zero represents ‘no discomfort’ and 10 ‘maximal discomfort’.

##### Perceived Exertion and General Feelings

Up to 30 min after the cessation of each RT protocol, participants rated their perceived exertion and general feelings for the entire session (via Qualtrics) using the modified category ratio rating of perceived exertion (RPE CR-10) scale [36, 37] and the feeling scale [38], respectively. Participants were asked ‘*how hard was your workout*?’ and to rate their perceived exertion for the session on a 0–10 (CR-10) scale whereby zero represents ‘rest’ and 10 ‘maximal exertion’. Participants were also asked *‘how do you currently feel’* and to assess their general feelings towards the session with the feeling scale, ranging from ‘ + 5’, which refers to ‘very good’, to ‘-5’, which refers to ‘very bad’ [38].

##### Perceived Recovery and Muscle Soreness

Participants rated their perceived level of recovery and muscle soreness 24 and 48 h after the completion of each experimental trial. The perceived recovery status (PRS) scale was used to assess the perceived level of recovery and involves a rating of perceived recovery between 0 and 10, with 0–2 representing very poor recovery with an expected decline in performance, 4–6 representing low-to-moderate recovery with an expected similar performance, and 8–10 representing high perceived recovery with an expected increase in performance [39, 40]. Participants were also asked to rate pain/soreness sensations in muscles of the chest (following three BP repetitions with the minimum possible load) from 0 to 10, whereby 0–1 represents little to no pain, 2 represents slight pain, 3–4 represents mild pain, 5–6 represents moderate pain, 7–8 represents severe pain, and 9–10 indicates the worst pain the individual has previously experienced following resistance training [39].

### Statistical Analysis

All statistical analyses were performed using ‘R’ software (v 4.0.2; R Core Team, https://www.r-project.org/). Two separate linear mixed models (with two-way interaction effects including ‘protocol’ and ‘time’ or ‘sex’, and ‘participant’ as a random effect) were generated (with the ‘lme4’ package in R) to first analyse differences between protocols at each timepoint (protocol × time), and secondly, differences between sexes for each protocol (protocol × sex), for the following outcome measures: (i) change in lifting velocity from pre-exercise to post-exercise, (ii) loss of lifting velocity (mean of entire set) from the first to the final set, and (iii) decrease in the total number of repetitions performed from the first to the final set. A linear mixed model (with ‘protocol’ and ‘time’ as fixed effects, and ‘participant’ as a random effect) was also used to assess differences between each RT protocol for all subjective measures (i.e. perceived discomfort, recovery, muscle soreness, exertion, and general feelings) at each time point measured. Diagnostic tests for each linear mixed model were performed using the ‘redres’ package in R to assess the validity of the model results. If model assumptions were violated, data were either log-transformed or analysed using nonparametric alternatives (i.e. Friedman’s test). Statistical significance was set at *P* =  < 0.05. Effect sizes (Cohen’s d) for within-protocol changes in outcome measures, and between-protocol differences in these changes, were calculated using the ‘effsize’ package in R with a Hedge’s g correction applied. The magnitude of effect size values was interpreted as < 0.2 = trivial, 0.2 to < 0.5 = small, 0.5 to < 0.8 = moderate, and ≥ 0.8 = large [41]. Post hoc analyses for pairwise comparisons were conducted when a main or interaction effect was statistically significant using Tukey’s test (or a Wilcoxon rank-sum test). To *complement* traditional null hypothesis significance testing, we also considered the outcomes based on the magnitude of effect size estimates and the associated 95% confidence interval width.

## Results

Descriptive characteristics (including total repetitions and lifting velocities) for each RT protocol are reported in Table 2 for males and females separately. All participants completed 100% of the procedures required in each experimental trial.

**Table 2:** Descriptive characteristics for each RT protocol

Variable	Men (*n* = 12)			Females (*n* = 12)		
	3-RIR	1-RIR	FAIL	3-RIR	1-RIR	FAIL
Total reps	44 ± 7	49 ± 8	45 ± 8	59 ± 12	65 ± 12	64 ± 14
Reps (first set)	9 ± 2	11 ± 2	12 ± 2	12 ± 3	14 ± 3	16 ± 3
Reps (final set)	6 ± 2*	6 ± 1*	5 ± 1*	9 ± 2*	9 ± 2*	8 ± 2*
*% Decrease Reps*	*29%*	*43%*	*59%*	*25%*	*37%*	*51%*
Mean LV (first set)	0.36 ± 0.05	0.33 ± 0.03	0.33 ± 0.04	0.35 ± 0.04	0.33 ± 0.04	0.32 ± 0.04
Mean LV (final set)	0.33 ± 0.05	0.30 ± 0.04*	0.23 ± 0.03*	0.34 ± 0.04	0.29 ± 0.05	0.26 ± 0.05*
*% Decrease LV*	*8.1%*	*11.3%*	*28.8%*	*1.1%*	*10.1%*	*19.6%*
Mean LV (last rep)	0.25 ± 0.04	0.19 ± 0.03	0.14 ± 0.03	0.23 ± 0.04	0.16 ± 0.03	0.12 ± 0.03

Data shown are presented as mean ± SD. Lifting velocity values represent mean velocity of the concentric portion of a repetition (m s^−1^).

*Denotes a statistically significant within-protocol difference from the first set.

LV, lifting velocity; reps, repetitions.

### Total Volume

**Fig. 2:**
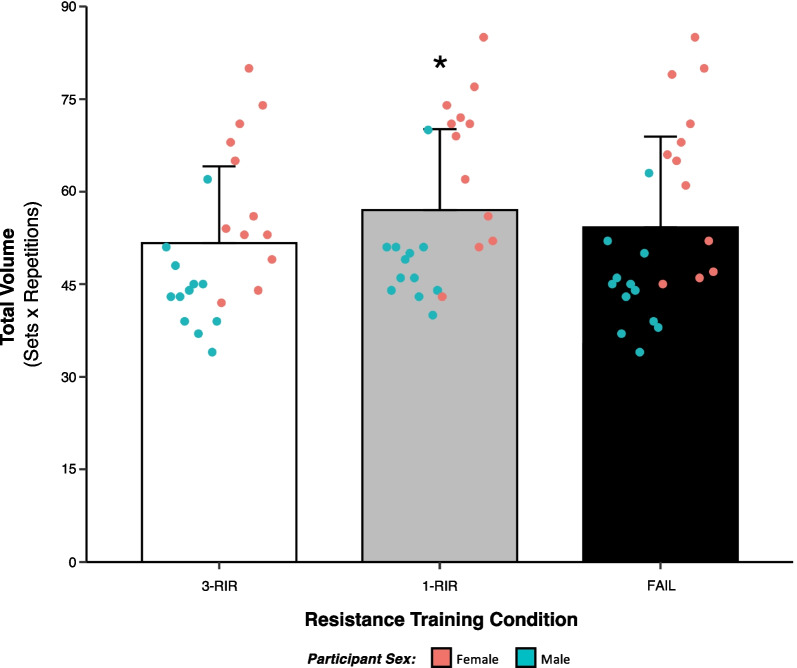
Influence of proximity-to-failure on total volume completed. Total volume calculated as the number of repetitions performed across six sets for each protocol (sets × repetitions). Data shown are presented as both protocol means (± SD) and individual values. *Denotes a statistically significant difference from FAIL and 3-RIR

A statistically significant effect of protocol on total volume (sets × reps) performed [*F* (2) = 12.32, *P* =  < 0.001] was found, with greater total volume achieved in 1-RIR versus both FAIL [ES = 0.18 (95% CI: 0.06, 0.30), *P* = 0.015] and 3-RIR [ES = 0.40 (95% CI: 0.21, 0.60), *P* =  < 0.001], but there was no statistically significant difference between FAIL and 3-RIR [ES = 0.18 (95% CI: -0.03, 0.39), *P* = 0.117] (Fig. 2). Further, there was a statistically significant effect of sex on total volume (mean of all protocols combined) [*F* (1) = 17.80, *P* =  < 0.001], with females performing more total volume than males [ES = 1.58 (95% CI: 1.04, 2.11), *P* =  < 0.001] (Table 2). No statistically significant interaction effect of protocol x sex was found (see Additional file 1: File S1 for all results).

### Recovery Time Course (Changes in Lifting Velocity from Pre-Exercise to Post-Exercise)

**Fig. 3:**
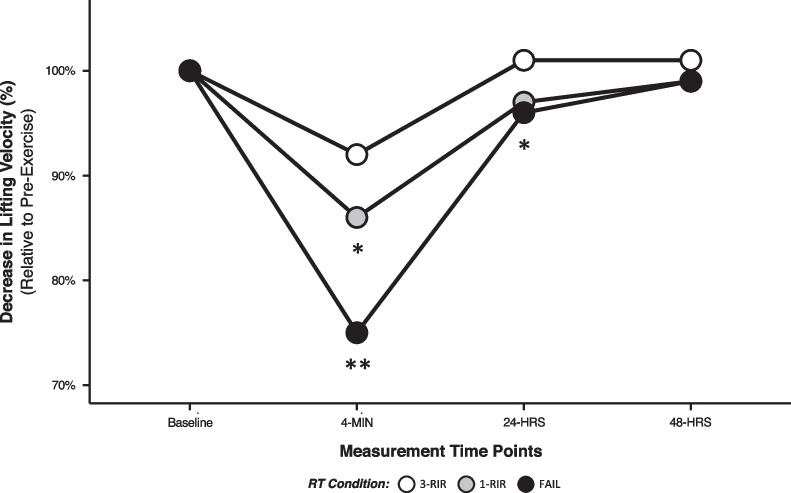
Post-exercise recovery time course of neuromuscular fatigue for all participants (males and females combined). Changes in lifting velocity are expressed as percentage values relative to pre-exercise. Data shown are presented as mean values (accompanying SD values can be found in Table 3). *Denotes a statistically significant difference from 3-RIR. **Denotes a statistically significant difference from 1-RIR and 3-RIR

**Fig. 4:**
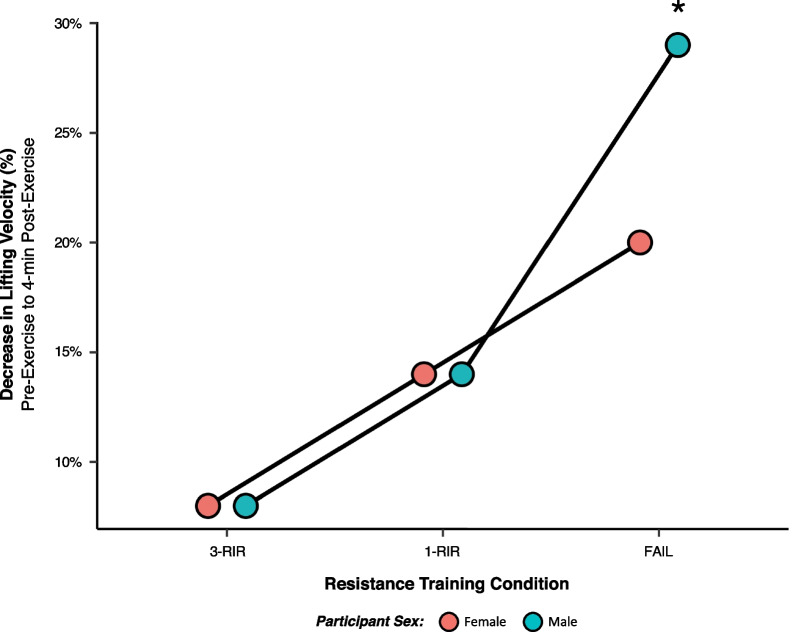
Relationship between proximity-to-failure and acute neuromuscular fatigue. Data shown are expressed as a percentage decrease in lifting velocity with a fixed load from pre-exercise to 4 min post-exercise (displayed as a positive value to indicate an increase in neuromuscular fatigue) in response to six sets performed on the barbell bench press exercise to either momentary muscular failure (FAIL), 1-RIR, or 3-RIR. Accompanying SD values can be found in Table 3. *Denotes a statistically significant difference from female participants

Statistically significant main and interaction effects of protocol [*F* (2) = 52.81, *P* =  < 0.001], time [*F* (2) = 229.58, *P* =  < 0.001], and protocol x time [*F* (4) = 18.18, *P* =  < 0.001] for the decrease in lifting velocity from pre-exercise to post-exercise were found (see Additional file 1: S2 for all results). The greatest decreases in lifting velocity from pre-exercise to post-exercise were observed at the 4-min time point for FAIL versus 1-RIR [ES = 1.16 (95% CI: 0.68, 1.63), *P* =  < 0.001] and 3-RIR [ES = 1.87 (95% CI: 1.26, 2.47), *P* =  < 0.001], and for 1-RIR versus 3-RIR [ES = 1.26 (95% CI: 0.80, 1.73), *P* =  < 0.001] (Table 3, Fig. 3). Greater decreases in lifting velocity from pre-exercise to post-exercise were also identified at 24 h for FAIL versus 3-RIR [ES = 0.90 (95% CI: 0.47, 1.32), *P* = 0.001], and 1-RIR versus 3-RIR [ES = 1.02 (95% CI: 0.40, 1.64), *P* = 0.001], but no statistically significant differences were identified at 48 h (Table 3, Fig. 3). To investigate sex differences at 4 min post-exercise, linear mixed modelling produced a statistically significant interaction effect of protocol × sex [*F* (2) = 7.14 *P* = 0.001], with post hoc analysis revealing a greater decrease in lifting velocity from pre-exercise to 4 min post-exercise in males versus females only when RT was performed to FAIL [ES = 0.82 (95% CI: -0.03, 1.67, *P* = 0.007], but no other statistically significant sex differences were found (Fig. 4).

**Table 3:** Mean decreases in lifting velocity from pre-exercise to post-exercise

	Post-exercise time point		
Protocol	4 min	24 h	48 h
3-RIR	− 0.05 ± 0.03	0.01 ± 0.03	0.01 ± 0.02
1-RIR	− 0.09 ± 0.03*	− 0.02 ± 0.03	− 0.01 ± 0.02
FAIL	− 0.15 ± 0.06*	− 0.02 ± 0.04	0.00 ± 0.04
*Male participants*			
3-RIR	− 0.04 ± 0.02	0.01 ± 0.02	0.01 ± 0.03
1-RIR	− 0.08 ± 0.03*	− 0.01 ± 0.02	− 0.01 ± 0.03
FAIL	− 0.17 ± 0.05*	− 0.02 ± 0.03	0.00 ± 0.04
*Female participants*			
3-RIR	− 0.05 ± 0.03	0.01 ± 0.03	0.00 ± 0.02
1-RIR	− 0.09 ± 0.03*	− 0.03 ± 0.03	− 0.01 ± 0.01
FAIL	− 0.12 ± 0.06*	− 0.02 ± 0.04	− 0.01 ± 0.05

Mean change calculated as ‘time point value’ minus ‘pre-exercise value’, with positive numbers indicating increases in lifting velocity (m s^−1^) from pre-exercise (and negative values indicate a decrease)

*Denotes a statistically significant within-protocol difference from pre-exercise to post-exercise

Data shown are presented as mean ± SD

### Loss of Lifting Velocity from First to Final Set

**Fig. 5:**
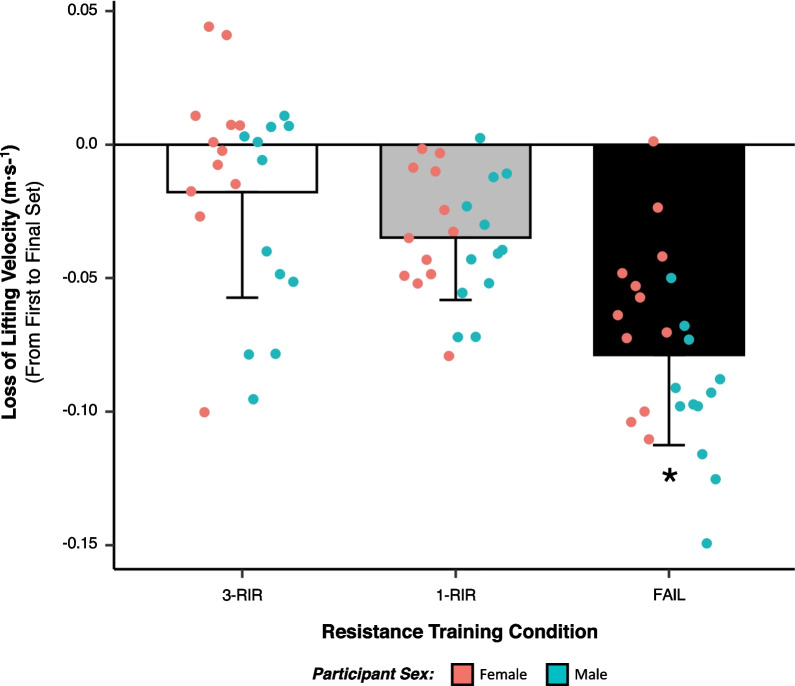
Loss of lifting velocity from first to final set. Data shown are presented as absolute values (m s^−1^) and as both protocol means (± SD) and individual values. *Denotes a statistically significant difference from 1-RIR and 3-RIR

Statistically significant main effects of protocol [*F* (2) = 30.14, *P* =  < 0.001] and sex [*F* (1) = 6.33, *P* = 0.012] were found for the loss of lifting velocity from the first set to the final set, but there was no interaction effect of protocol x sex (see Additional File 1: S3 for all results). Post hoc analysis of decreases in lifting velocity from the first set to the final set for each protocol (Mean ± *SD*: FAIL = − 0.08 ± 0.03, 1-RIR = − 0.03 ± 0.02, 3-RIR = − 0.02 ± 0.04) revealed greater decreases for FAIL versus both 1-RIR [ES = 1.46 (95% CI: 0.63, 2.29), *P* =  < 0.001] and 3-RIR [ES = 1.59 (95% CI: 1.02, 2.16), *P* =  < 0.001] (Fig. 5). Further post hoc analysis of sex also revealed a greater decrease in lifting velocity (mean of all protocols combined) from the first set to the final set (Mean ± *SD*: Male = − 0.05 ± 0.04, Female = − 0.03 ± 0.04) for male versus female participants [ES = 0.52 (95% CI: 0.06, 1.00), *P* = 0.020], with the largest effect size differences between male and female participants found for FAIL [ES = 1.09 (95% CI: 0.21, 1.96)] and 3-RIR [ES = 0.66 (95% CI: 0.18, 1.50)].

### Repetition Loss from First to Final Set

**Fig. 6:**
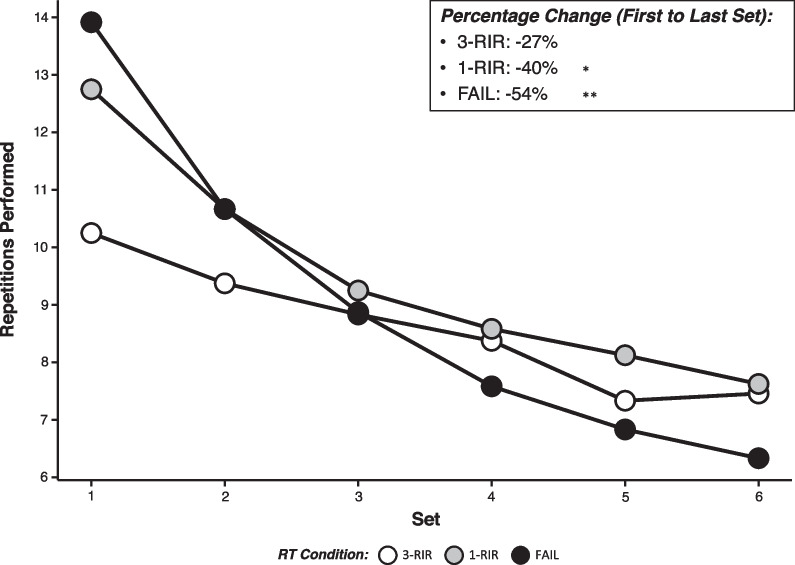
Number of repetitions performed in each set. Data shown are presented as mean (absolute) values (accompanying SD values can be found in Additional file 1: S4). *Denotes a statistically significant difference from 3-RIR. **Denotes a statistically significant difference from 1-RIR and 3-RIR

A statistically significant main effect of protocol [*F* (2) = 64.96, *P* =  < 0.001] for repetition loss from the first set to the final set was found, but there was no main effect of sex or interaction effect of protocol x sex (see Additional file 1: S4 for all results). Post hoc analysis of the decrease in repetitions performed from the first set to the final set for each protocol (Mean ± *SD*: FAIL = − 7.58 ± 1.89, 1-RIR = − 5.13 ± 1.73, 3-RIR = − 2.79 ± 1.84) revealed greater decreases for FAIL versus both 1-RIR [ES = 1.31 (95% CI: − 0.78, 1.84), *P* =  < 0.001] and 3-RIR [ES = 2.49 (95% CI: 1.67, 3.30), *P* =  < 0.001], and repetition loss was greater for 1-RIR versus 3-RIR [ES = 1.26 (95% CI: 0.56, 1.97), *P* =  < 0.001]. The decrease in repetitions from set-to-set for all protocols is shown in Fig. 6.

### Perceived Discomfort, Perceived Exertion, and General Feelings

**Fig. 7:**
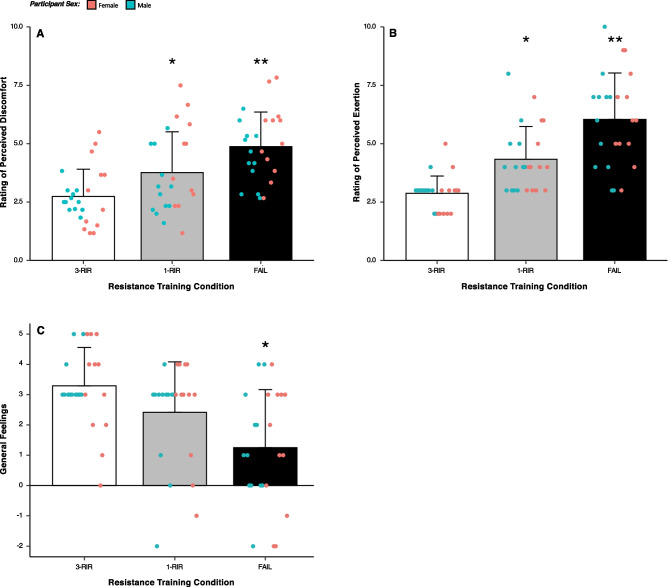
Ratings of post-set perceived discomfort (**A**), post-exercise perceived exertion (**B**), and post-exercise general feelings (**C**). Data shown are presented as mean ± SD. *Denotes a statistically significant difference from 3-RIR condition. **Denotes a statistically significant difference from 1-RIR and 3-RIR conditions

A statistically significant main effect of protocol for rating of perceived discomfort [*Chi* (2) = 30.98, *P* =  < 0.001], rating of perceived exertion [*Chi* (2) = 35.89 *P* =  < 0.001], and general feelings using the feeling scale was found [*Chi* (2) = 17.13, *P* =  < 0.001]. Post hoc analysis revealed that ratings of perceived discomfort were greater for FAIL versus both 1-RIR [ES = 0.65 (95% CI: 0.36, 0.94), *P* = 0.001] and 3-RIR [ES = 1.50 (95% CI: 1.06, 1.93), *P* =  < 0.001], and greater for 1-RIR versus 3-RIR [ES = 0.62 (95% CI: 0.26, 0.99), *P* = 0.005]. Further, ratings of perceived exertion were greater for FAIL versus both 1-RIR [ES = 0.95 (95% CI: 0.37, 1.53), *P* = 0.003] and 3-RIR [ES = 1.85 (95% CI: 1.12, 2.57), *P* =  < 0.001], and greater for 1-RIR versus 3-RIR [ES = 1.14 (95% CI: 0.66, 1.63), *P* =  < 0.001]. Lastly, lower feeling scale ratings were observed for FAIL versus 3-RIR [ES = -1.19 (95% CI: − 1.81, − 0.58), *P* = 0.001] and for 1-RIR versus 3-RIR [ES = − 0.56 (95% CI: − 1.00, − 0.12), *P* = 0.025]. Figure 7 displays mean, standard deviation, and individual values for perceived discomfort, perceived exertion, and feeling scale ratings (see Additional file 1: S5-S7 for all results).

### Perceived Recovery and Muscle Soreness

**Fig. 8:**
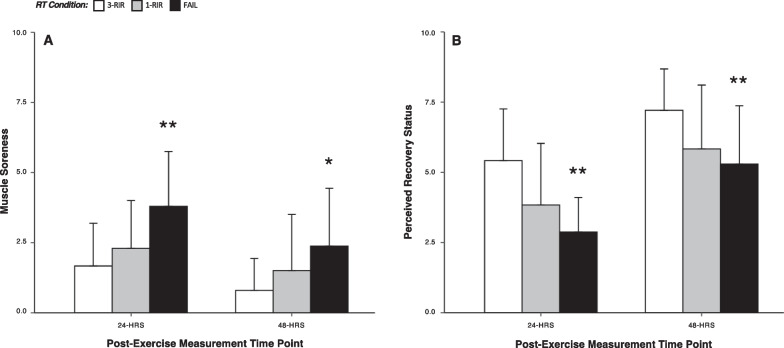
Ratings of muscle soreness (**A**) and perceived recovery status (**B**) at 24 and 48 h post-exercise. Data shown are presented as mean ± SD. *Denotes a statistically significant difference from 3-RIR. **Denotes a statistically significant difference from 1-RIR and 3-RIR

A statistically significant main effect of protocol for ratings of muscle soreness at 24 h [*Chi* (2) = 18.40, *P* =  < 0.001] and 48 h [*Chi* (2) = 14.08, *P* =  < 0.001] and perceived recovery status at 24 h [*Chi* (2) = 21.30, *P* =  < 0.001] and 48 h was found [*Chi* (2) = 12.83, *P* = 0.002]. Post hoc analysis revealed greater muscle soreness ratings at 24 h post-exercise for FAIL versus both 1-RIR [ES = 0.79 (95% CI: 0.19, 1.39), *P* = 0.023] and 3-RIR [ES = 1.16 (95% CI: 0.64, 1.68), *P* =  < 0.001], and greater perceived recovery ratings at 24 h post-exercise for 3-RIR versus FAIL [ES = 1.55 (95% CI: 0.83, 2.27), *P* =  < 0.001], and for 3-RIR versus 1-RIR [ES = 0.75 (95% CI: 0.21, 1.29), *P* = 0.014]. Further post hoc analysis revealed greater muscle soreness ratings at 48 h post-exercise for FAIL versus 3-RIR [ES = 0.90 (95% CI: 0.31, 1.50), *P* = 0.004], and greater perceived recovery ratings at 48 h post-exercise for 3-RIR versus FAIL [ES = 1.02 (95% CI: 0.45, 1.58), *P* = 0.003] and for 3-RIR versus 1-RIR [ES = 0.66 (95% CI: 0.25, 1.06), *P* = 0.008]. Figure 8 displays mean and standard deviation values for muscle soreness and perceived recovery ratings at 24 h and 48 h (see Additional file 1: S8-S9 for all results).

## Discussion

### Influence of Proximity-to-Failure on Neuromuscular Fatigue

Our primary findings suggest that (i) *acute* neuromuscular fatigue (i.e. decreases in lifting velocity from pre-exercise to 4 min post-exercise, and from the first to the final set) increases in resistance-trained males and females as proximity-to-failure nears and is greatest when momentary muscular failure is reached, providing evidence for a *linear* relationship between proximity-to-failure and acute neuromuscular fatigue, (ii) 48 h is likely sufficient for complete recovery of neuromuscular function when RT is performed for six sets on the barbell bench press, independent of the proximity-to-failure reached, (iii) performing RT to 3-RIR may be a viable strategy to minimise the neuromuscular fatigue incurred from RT and potentially improve RT performance at 24 and 48 h post-exercise, and (iv) males experience greater acute neuromuscular fatigue than females when RT is performed to momentary muscular failure.

Although previous research suggests that neuromuscular fatigue is greater following RT performed to momentary muscular failure versus non-failure [15, 42–46], considering the ambiguity and variability in the proximity-to-failure achieved during non-failure RT protocols, these data are unable to inform the specific effect of different proximities-to-failure on neuromuscular fatigue. To address this research limitation, we employed subjective RIR prediction to control the proximity-to-failure reached by participants in our 1-RIR and 3-RIR protocols that were compared with RT performed to momentary muscular failure (FAIL). As proximity-to-failure neared, we observed a graded increase in acute neuromuscular fatigue at 4 min post-exercise (Fig. 3) and from the first to the final set (Fig. 5), with the highest levels of neuromuscular fatigue found when participants performed RT to FAIL versus 1-RIR and 3-RIR (FAIL > 1-RIR > 3-RIR). These results corroborate previous findings that showed greater decreases in lifting velocity immediately post-exercise as sets were terminated with higher magnitudes of velocity loss (and therefore as proximity-to-failure neared) [12]; however, the magnitude of velocity loss used to control set termination cannot be accurately translated to RIR [1]. As such, our data provide novel insights into the specific effect of reaching different proximities-to-failure during RT, quantified via RIR, on neuromuscular fatigue. Of interest are the *central* (i.e. suppression of skeletal muscle excitation by the central nervous system) and *peripheral* (i.e. energy depletion and intramuscular perturbations in metabolite concentration and calcium (Ca^+2^) kinetics that impair cross-bridge formation) mechanisms underpinning the neuromuscular fatigue observed [47, 48], which may suppress (i) force production by type II muscle fibres and their exposure to mechanical tension during RT (potentially explaining the *non-linear* relationship between proximity-to-failure and muscle hypertrophy [49]), and (ii) the absolute load lifted on a given exercise, ultimately hampering muscle hypertrophy or maximal strength development, respectively. Our results also demonstrate that contrary to our hypothesis, the majority of participants experienced complete recovery of neuromuscular function at 24 h post-exercise, independent of the RT protocol completed (Table 3); however, it is possible that increasing the number of sets performed for a given exercise or muscle group may elongate the recovery time course of neuromuscular function. Although not statistically significant (*P* =  > 0.05), we observed a slight increase in lifting velocity, and thus improvement in neuromuscular function, at 24 h post-exercise for 3-RIR (Fig. 3), which was not evident for FAIL and 1-RIR. Overall, these data provide evidence for a linear relationship between proximity-to-failure and acute neuromuscular fatigue and suggest that performing RT to 3-RIR incurs low levels of neuromuscular fatigue that has minimal negative effects on force production 24 and 48 h post-exercise, while inducing a possible ‘supercompensation’ (or potential ‘priming’) effect.

To provide further insights into the neuromuscular fatigue incurred from each RT protocol, we also assessed the number of repetitions performed in each of the six sets completed and the total volume (sets × reps) achieved. Similar to previous research comparing RT performed to momentary muscular failure versus 20% velocity loss [45], we also found that FAIL resulted in the highest number of repetitions performed in the first set (14 ± 3) compared to 1-RIR (13 ± 3) and 3-RIR (10 ± 3), but fewer repetitions were performed in the final set (FAIL = 6 ± 2, 1-RIR = 8 ± 2, 3-RIR = 7 ± 2), leading to a percentage loss in repetitions from the first to the final set of 54% for FAIL versus 40% and 27% for 1-RIR and 3-RIR, respectively (Fig. 6). While FAIL resulted in the most repetitions in the first set, decreases in repetitions performed with a given load likely reflect a suppressed force production and an overall decrease in the exposure of active muscle fibres to mechanical tension across the multiple subsequent sets, highlighting the *possibility* of a similar hypertrophic stimulus achieved between our RT protocols with differing levels of neuromuscular fatigue. Indeed, differences in repetitions performed per set across our RT protocols resulted in a similar total volume achieved for FAIL (54 ± 15) and 3-RIR (52 ± 12), with the greatest total volume observed for 1-RIR (57 ± 13), suggesting that RT volume may be maximised in multiple-set protocols when terminating sets close to (i.e. ~ 1-RIR), but prior to, momentary muscular failure (Fig. 2). These data, in corroboration with similar results reported elsewhere (for example: Mangine et al. [15] showed no statistically significant difference in total volume achieved over five sets between RT performed to momentary muscular failure and ‘0–3-RIR’), suggest that the proximity-to-failure reached across multiple sets has a major influence on the total volume accumulated during RT, potentially influencing subsequent physiological adaptations that may be associated with the total RT volume achieved.

#### Role of Biological Sex in the Influence of Proximity-to-Failure on Neuromuscular Fatigue

To elucidate potential differences in neuromuscular fatigue between biological sexes, both male and female participants with a similar level of RT experience (Table 1) were recruited for this study. Our analysis of sex differences revealed that FAIL induced greater *acute* neuromuscular fatigue at 4 min post-exercise in males compared to females (Fig. 4); however, no sex differences in neuromuscular fatigue were found for 1-RIR and 3-RIR. When analysing the mean of all RT protocols combined, we also found males experienced greater loss of lifting velocity from the first to the final set compared to females [with the greatest effect sizes observed for FAIL (ES = 1.09) and 3-RIR (ES = 0.66)], providing further evidence for the influence of biological sex on *acute* neuromuscular fatiguability during RT (Fig. 5). Explaining these potential sex differences in neuromuscular fatiguability may be the greater absolute load lifted by males compared to females in our study [50], however, it is also possible that the degree of arterial occlusion experienced during RT contributed to sex differences in neuromuscular fatigability, with males possessing larger muscle mass than females and likely experiencing more arterial occlusion [51–53] throughout an RT set performed to momentary muscular failure. Further, females may have experienced more recovery within the 4 min rest period allocated in-between sets than males due to having a greater proportion of type I skeletal muscle fibres [54–56] comprising a high capillary density and allowing for greater vasodilation and muscle perfusion [51–53]. Any of these factors, alone or in combination, could have ultimately resulted in the male participants experiencing greater *acute* neuromuscular fatigue over multiple sets when momentary muscular failure was reached and the inter-set recovery period was confined to 4 min. Similar to our results, recent research [57] found greater acute neuromuscular fatigue in males compared to females when RT was performed to a 40% versus 20% velocity loss threshold, although this sex difference was absent following the completion of an 8-week RT intervention. In contrast, another study [46] found greater neuromuscular fatigue up to 72 h post-exercise in males compared to females when RT was performed for five repetitions with 80% 1-RM, but no sex differences were found when RT was performed to momentary muscular failure; however, considering inconsistencies in lifting velocity data, it is possible that participants in this study were not well familiarised to performing RT with maximal intended lifting velocity, a necessary requirement to obtain reliable and valid measures of lifting velocity. Nonetheless, considering the set termination methods applied in these studies [46, 57] are unable to inform RIR values, the present findings provide unique insights into the potential interaction of proximity-to-failure with biological sex, revealing possible sex differences in neuromuscular fatigue.

### Perceptual Measures of Neuromuscular Fatigue

To evaluate differences in perceptual responses between RT protocols, we assessed ratings of perceived discomfort immediately after each set performed, session ratings of perceived exertion, general feelings within 30 min of exercise cessation, and ratings of perceived muscle soreness and recovery 24 and 48 h post-exercise. We found that (i) perceived discomfort and exertion increased gradually as proximity-to-failure neared, (ii) general feelings following RT were similar for FAIL and 1-RIR, but worse for FAIL and 1-RIR compared to 3-RIR, (iii) perceived muscle soreness was greater for FAIL versus 3-RIR at both 24 and 48 h post-exercise, but was only greater for FAIL versus 1-RIR at 24 h post-exercise, and (iv) perceived recovery was lower for FAIL versus both 1-RIR and 3-RIR at both 24 and 48 h post-exercise.

Perceptual responses are important considerations when prescribing RT as they may influence the affective response to RT and subsequent exercise adherence, and ultimately, physiological adaptations to RT. In support of previous research [45, 58], we found that perceived discomfort and exertion increased gradually as proximity-to-failure neared (Fig. 7A and 7B), with the greatest ratings observed for FAIL (FAIL > 1-RIR > 3-RIR). Although ratings of perceived discomfort and exertion were 5 ± 1 and 6 ± 2 for FAIL, respectively, these results should be interpreted within the context of an RT session involving numerous exercises, whereby ratings of perceived discomfort and exertion may be even higher. It is also possible that perceived discomfort would be greater if the relative load lifted was lower (and thus the repetitions per set higher) [35] or the exercise performed involved a larger amount of active musculature (e.g. leg press versus bench press). Additionally, although general feelings following RT were similar for FAIL and 1-RIR, the ratings were lower compared to 3-RIR (Fig. 7C), providing further support for the idea that exercise difficulty may be a primary influencer of the affective response to an exercise bout [59, 60], which may be linked to long-term exercise adherence [61]. There is, however, large intra-individual variability in feelings towards a given RT protocol; for example, feeling scale ratings ranged from -3 (‘fairly bad’) to + 3 (‘good’) following RT to FAIL. As such, these results suggest that an individual’s affective valence, along with their perceptions of discomfort and exertion, should be considered when prescribing proximity-to-failure during RT.

In combination with our objective measures of lifting velocity to assess neuromuscular fatigue, we also assessed perceptual measures of recovery 24 and 48 h post-exercise. Contrasting previous research [62] that found no significant difference in perceived muscle soreness between RT performed to set failure (definition other than momentary muscular failure) versus non-failure, we found perceived muscle soreness was greater for FAIL versus 3-RIR at both 24 and 48 h post-exercise, but was only greater for FAIL versus 1-RIR at 24 h post-exercise (Fig. 8A). Although perceived muscle soreness was still present 48 h post-exercise for all RT protocols, this did not seem to negatively influence the recovery of neuromuscular function (assessed via changes in lifting velocity) at 48 h post-exercise (Fig. 3). Similar results were also found for perceived recovery status, with lower ratings for FAIL versus both 1-RIR and 3-RIR at both 24 and 48 h post-exercise (Fig. 8B); however, the level of perceived recovery did not always reflect lifting velocity outcomes. For example, five participants expected ‘declined performance’ 24 h following RT to 1-RIR, but instead experienced complete recovery of lifting velocity. Although our findings suggest perceptions of muscle soreness and recovery may not always reflect objective changes in lifting velocity in a research setting, in practice (when individuals may not be prompted to perform RT maximally by qualified supervisors), these perceptions may influence performance and should be considered during RT prescription. Given our previous scoping review [1] found only two studies [45, 62] investigating the influence of proximity-to-failure on perceptual responses to RT, the present findings provide unique insights into the influence of proximity-to-failure on these measures and may have important implications for enjoyment with, and potentially long-term adherence to, RT.

### Practical Application of Key Findings

When multiple exercises for a given muscle group are performed in an RT session, various proximities-to-failure can be employed to limit large decrements in force production that may accumulate over multiple sets and may negatively influence subsequent physiological adaptations. Considering set-volume (i.e. the number of sets performed to, or close to momentary muscular failure per muscle group per week [63]) may also influence the level of neuromuscular fatigue incurred (given that we observed decreases in repetitions performed across sets in all three conditions), the proximities-to-failure achieved during RT should also be dependent on the set-volume assigned, with closer proximities-to-failure reached when (i) lower set-volumes or (ii) a longer time course of recovery between RT sessions (e.g. 48–72 h) involving the same muscle group are employed. The decision to reach momentary muscular failure should be primarily based on safety, the prescription of other RT variables (e.g. set-volume, frequency, exercise order) and the affective valence of an individual; for example, some individuals may experience a negative affective response or high levels of perceived discomfort and exertion when reaching momentary muscular failure, but other individuals may not. Potential sex differences in neuromuscular fatigability should also be considered when prescribing proximity-to-failure during RT, and based on our findings, we also suggest that i) males do not perform sets to momentary muscular failure as frequently as females, and ii) if momentary muscular failure is reached, males employ longer post-set rest periods than females. However, these recommendations are based on group average responses, and considering some female participants in the present study appeared to show greater fatigability than some male participants (Fig. 5), individual fatigability should still be considered for RT prescription; for example, participants that were highly fatigable experienced greater decreases in repetitions from set-to-set, and this can be used as an indicator of individual fatigability in practice. Notably, although perceived recovery and muscle soreness may also inform individual fatigability and the degree of recovery experienced in-between RT sessions, it is important to consider that these perceptions may not always reflect the objective performance capabilities of an individual.

### Limitations of Current Research and Future Directions

The present study assessed neuromuscular fatigue with the barbell bench press exercise, but whether our results can be generalised to other exercises and/or muscle groups is unclear. Future research should thus investigate the potential effect of other exercises and/or muscle groups on neuromuscular fatigue in response to different proximities-to-failure to provide further insights that may improve practical RT prescription. Considering our RT protocols involved subjective RIR prediction, whether participants terminated their sets accurately, as per the RIR target, is unknown. However, we employed an extended familiarisation that required participants to perform RT to momentary muscular failure and subjectively predict a 1- and 3-RIR on two separate occasions (Sect. ‘Pre-Visit Sessions’), to theoretically increase the accuracy of their RIR predictions. Previous research has also shown that the accuracy of RIR predictions increases with RT experience [22, 23], and a recent meta-analysis [64] found individuals typically underpredict RIR by approximately one repetition, independent of RT experience. Considering the lack of insight into the specific effect of proximity-to-failure on neuromuscular fatigue throughout the available literature, future research should consider employing subjective RIR prediction to control set termination whilst ensuring that: (i) participants are provided with unambiguous instructions and are well familiarised with the procedures before commencing experimental trials, (ii) higher-loads (e.g. > 50% 1-RM) are used versus lower-loads, and (iii) if safe to do so, momentary muscular failure is first experienced on a given exercise to ‘anchor’ subjective perceptions of proximity-to-failure [1]. Finally, our analysis of neuromuscular fatigue is also limited to the outcome measure tested (e.g. changes in lifting velocity), and as such, future research should combine measures of lifting velocity with other objective measures of neuromuscular fatigue such as maximum voluntary isometric contraction and twitch interpolation to provide insight into both central and peripheral mechanisms of neuromuscular fatigue.

## Conclusion

In resistance-trained males and females, we observed greater decreases in lifting velocity on the barbell bench press exercise from pre-exercise to 4 min post-exercise and from the first to the final set performed as proximity-to-failure neared (FAIL > 1-RIR > 3-RIR), providing evidence for a *linear relationship* between proximity-to-failure and *acute* neuromuscular fatigue. Further, when momentary muscular failure was reached (FAIL), males also experienced greater acute neuromuscular fatigue than females. A slight decrement in neuromuscular function when RT was performed to momentary muscular failure and 1-RIR was sustained at 24 h post-exercise versus 3-RIR, with 48 h of recovery post-exercise likely sufficient for complete recovery of neuromuscular function when RT is performed for six sets on the barbell bench press exercise, independent of the proximity-to-failure reached. Our assessments of the perceptual response to RT also showed that as proximity-to-failure neared, ratings of perceived discomfort, exertion, and muscle soreness increased, general feelings worsened, and perceived recovery decreased. Overall, proximity-to-failure not only influences the neuromuscular fatigue incurred from RT, but is also a key determinant of the perceptual responses to RT.

## Supplementary Information

**Additional file 1:** Supplementary document containing data generated from statistical analyses.

## Data Availability

The data sets used and/or analysed during the current study are available from the corresponding author upon reasonable request.

## Abbreviations

BP: Bench press
Ca^+2^: Calcium
CI: Confidence interval
ES: Effect size
FS: Feeling scale
KG: Kilograms
LV: Lifting velocity
MS: Muscle soreness
1-RM: One repetition maximum
PRS: Perceived recovery status
P/W: Per week
RIR: Repetitions-in-reserve
Reps: Repetitions
RT: Resistance training
RPD: Rating of perceived discomfort
RPE: Rating of perceived exertion
SD: Standard deviation
