# Supplementary material for: Influence of Resistance Training Proximity-to-Failure, Determined by Repetitions-in-Reserve, on Neuromuscular Fatigue in Resistance-Trained Males and Females
Source: Sports Med Open. 2023 Feb 8;9:10. doi: 10.1186/s40798-023-00554-y (PMC9908800; doi:10.1186/s40798-023-00554-y)
Supplement: Supplementary file 1 — Additional file 1: Supplementary document containing data generated from statistical analyses. [file 40798_2023_554_MOESM1_ESM.pdf]

Title: Influence of Resistance Training Proximity-to-Failure, Determined by Repetitions-in-Reserve, on Neuromuscular Fatigue in Resistance-Trained Males and Females.

Running title: Influence of Proximity-to-Failure on Neuromuscular Fatigue.

Authors: Martin C. Refalo[1], Eric R. Helms[2], D. Lee Hamilton[3], & Jackson J. Fyfe[3].

Affiliations:

1. Centre for Sport Research (CSR), School of Exercise and Nutrition Sciences, Deakin University, Geelong, Australia.
2. Sport Performance Research Institute New Zealand (SPRINZ), Auckland University of Technology, Auckland, New Zealand.
3. Institute for Physical Activity and Nutrition (IPAN), School of Exercise and Nutrition Sciences, Deakin University, Geelong, Australia.

Corresponding author:

Mr. Martin Refalo
Centre for Sport Research (CSR), School of Exercise and Nutrition Sciences
Deakin University, Geelong, Australia
Email: mrefalo@deakin.edu.au
ORCiD: 0000-0003-3755-6216

S1. Total Volume

1.1 Linear Mixed Effects Model (Protocol x Sex)

**Effect**	**DF**	***F*-Value**	***P*-Value**
Protocol	2	12.32	<0.001
Sex	1	17.80	<0.001
Protocol x Sex	2	1.59	0.204

1.1.1 Tukey's Pairwise Comparisons

**Protocol**	**ES (CI)**	***P*-Value**
1-RIR vs. 3-RIR	0.40 (0.21, 0.60)	<0.001
1-RIR vs. FAIL	-0.18 (-0.30, -0.06)	0.015
3-RIR vs. FAIL	0.18 (-0.03, 0.39)	0.117
**Sex**		
Male vs. Female	1.58 (1.05, 2.11)	<0.001

1.1.2 Descriptive Statistics [Total Volume (Sets x Repetitions)]

**Protocol**	**Mean**	***SD***
1-RIR	52	12
3-RIR	57	13
FAIL	54	15
**Sex**		
Males	46	7
Females	63	13
**Protocol x Sex**		
1-RIR *(Male)*	49	8
1-RIR *(Female)*	65	12
3-RIR *(Male)*	44	7
3-RIR *(Female)*	59	12
FAIL *(Male)*	45	8
FAIL *(Female)*	64	14

S2. Recovery Time-Course from Pre-Exercise to Post-Exercise

2.1 Linear Mixed Effects Model (Protocol x Time)

**Effect**	**DF**	***F*-Value**	***P*-Value**
Protocol	2	52.81	<0.001
Time	2	229.58	<0.001
Protocol x Time	4	18.18	<0.001

2.1.1 Tukey's Pairwise Comparisons

**Protocol**	**ES (CI)**	***P*-Value**
1-RIR vs. 3-RIR	0.66 (0.46, 0.85)	<0.001
1-RIR vs. FAIL	0.26 (0.10, 0.42)	<0.001
3-RIR vs. FAIL	0.58 (0.41, 0.74)	<0.001
**Time**		
24-hrs vs. 48-hrs	0.32 (0.11, 0.54)	0.067
24-hrs vs. 4-min	1.55 (1.24, 1.87)	<0.001
48-hrs vs. 4-min	1.83 (1.42, 2.25)	<0.001
**Protocol x Time**		
1-RIR vs. 3-RIR *(4-min)*	1.26 (0.80, 1.73)	<0.001
1-RIR vs. FAIL *(4-min)*	1.16 (0.68, 1.63)	<0.001
3-RIR vs. FAIL *(4-min)*	1.87 (1.26, 2.47)	<0.001
1-RIR vs. 3-RIR *(24-hrs)*	1.02 (0.40, 1.64)	0.001
1-RIR vs. FAIL *(24-hrs)*	0.01 (-0.52, 0.55)	0.998
3-RIR vs. FAIL *(24-hrs)*	0.90 (0.47, 1.32)	0.001
1-RIR vs. 3-RIR *(48-hrs)*	0.57 (0.08, 1.06)	0.189
1-RIR vs. FAIL *(48-hrs)*	-0.02 (-0.48, 0.43)	0.993
3-RIR vs. FAIL *(48-hrs)*	0.36 (-0.10, 0.83)	0.230

2.1.2 Descriptive Statistics (Decrease in Lifting Velocity from Pre-Exercise)

**Protocol**	**Mean**	***SD***
1-RIR	-0.04	0.04
3-RIR	-0.01	0.04

FAIL	-0.06	0.08
**Time**		
4-min	-0.09	0.06
24-hrs	-0.01	0.03
48-hrs	0.00	0.03
**Protocol x Time**		
1-RIR *(4-min)*	-0.09	0.03
3-RIR *(4-min)*	-0.05	0.03
FAIL *(4-min)*	-0.15	0.06
1-RIR *(24-hrs)*	-0.02	0.03
3-RIR *(24-hrs)*	0.01	0.03
FAIL *(24-hrs)*	-0.02	0.04
1-RIR *(48-hrs)*	-0.01	0.02
3-RIR *(48-hrs)*	0.01	0.02
FAIL *(48-hrs)*	0.00	0.04

2.2 Linear Mixed Effects Model [Protocol x Sex (4-min)]

Effect	DF	*F*-Value	*P*-Value
Protocol	2	89.57	<0.001
Sex	1	0.90	0.342
Protocol x Sex	2	7.14	0.001

2.2.1 Tukey's Pairwise Comparisons

Protocol x Sex	ES (CI)	*P*-Value
1-RIR *(Male vs. Female)*	0.10 (-0.72, 0.92)	0.834
3-RIR *(Male vs. Female)*	0.12 (-0.69, 0.94)	0.828
FAIL *(Male vs. Female)*	0.82 (-0.03, 1.67)	0.007

2.2.2 Descriptive Statistics (Decrease in Lifting Velocity from Pre-Exercise)

Protocol x Sex	Mean	*SD*
1-RIR *(Male)*	-0.08	0.03
1-RIR *(Female)*	-0.09	0.03
3-RIR *(Male)*	-0.04	0.02
3-RIR *(Female)*	-0.05	0.03
FAIL *(Male)*	-0.17	0.05
FAIL *(Female)*	-0.12	0.06

2.3 Within-Protocol Statistical Differences from Pre-Exercise

**Protocol x Time**	**ES (CI)**	***P*-Value**
**All Participants**		
1-RIR *(4-min)*	0.87 (0.71, 1.02)	0.003
1-RIR *(24-hrs)*	0.21 (0.09, 0.33)	0.453
1-RIR *(48-hrs)*	0.05 (-0.04, 0.15)	0.847
3-RIR *(4-min)*	0.55 (0.40, 0.69)	0.056
3-RIR *(24-hrs)*	-0.10 (-0.23, 0.03)	0.717
3-RIR *(48-hrs)*	-0.10 (-0.22, 0.02)	0.712
FAIL *(4-min)*	1.59 (1.20, 1.98)	<0.001
FAIL *(24-hrs)*	0.25 (0.08, 0.43)	0.365
FAIL *(48-hrs)*	0.05 (-0.15, 0.25)	0.848
**Male Participants**		
1-RIR *(4-min)*	0.78 (0.57, 0.99)	0.046
1-RIR *(24-hrs)*	0.87 (0.71, 1.02)	0.003
1-RIR *(48-hrs)*	0.21 (0.09, 0.33)	0.453
3-RIR *(4-min)*	0.47 (0.31, 0.62)	0.219
3-RIR *(24-hrs)*	-0.14 (-0.29, 0.02)	0.725
3-RIR *(48-hrs)*	-0.15 (-0.33, 0.03)	0.693
FAIL *(4-min)*	1.83 (1.35, 2.30)	<0.001
FAIL *(24-hrs)*	0.26 (0.04, 0.48)	0.501
FAIL *(48-hrs)*	0.03 (-0.21, 0.27)	0.945
**Female Participants**		
1-RIR *(4-min)*	0.84 (0.62, 1.06)	0.036
1-RIR *(24-hrs)*	0.33 (0.15, 0.52)	0.387
1-RIR *(48-hrs)*	0.06 (-0.02, 0.13)	0.883
3-RIR *(4-min)*	0.56 (0.32, 0.81)	0.150
3-RIR *(24-hrs)*	-0.05 (-0.24, 0.14)	0.885
3-RIR *(48-hrs)*	-0.04 (-0.18, 0.11)	0.919
FAIL *(4-min)*	1.32 (0.77, 1.86)	0.001
FAIL *(24-hrs)*	0.20 (-0.05, 0.45)	0.560
FAIL *(48-hrs)*	0.07 (-0.23, 0.38)	0.839

S3. Lifting Velocity Loss from First to Final Set

3.1 Linear Mixed Effects Model (Protocol x Sex)

Effect	DF	*F*-Value	*P*-Value
Protocol	2	30.14	<0.001
Sex	1	6.33	0.012
Protocol x Sex	2	1.66	0.190

3.1.1 Tukey's Pairwise Comparisons

Protocol	ES (CI)	*P*-Value
1-RIR vs. 3-RIR	0.50 (-0.05, 1.06)	0.101
1-RIR vs. FAIL	1.46 (0.63, 2.29)	<0.001
3-RIR vs. FAIL	1.59 (1.02, 2.16)	<0.001
**Sex**		
Male vs. Female	0.53 (0.06, 1.00)	0.020

3.1.2 Descriptive Statistics (Decrease in Lifting Velocity from First to Final Set)

Protocol	Mean	*SD*
1-RIR	-0.03	0.02
3-RIR	-0.02	0.04
FAIL	-0.08	0.03
**Sex**		
Males	-0.05	0.04
Females	-0.03	0.04
**Protocol x Sex**		
1-RIR *(Male)*	-0.04	0.02
1-RIR *(Female)*	-0.03	0.02
3-RIR *(Male)*	-0.03	0.04
3-RIR *(Female)*	0.00	0.04
FAIL *(Male)*	-0.10	0.03
FAIL *(Female)*	-0.06	0.03

3.2 Within-Protocol Statistical Differences from First Set to Final Set

Protocol	ES (CI)	*P*-Value
**All Participants**		
1-RIR	0.85 (0.58, 1.12)	0.003
3-RIR	0.39 (0.02, 0.76)	0.168
FAIL	1.88 (1.33, 2.43)	<0.001
**Male Participants**		
1-RIR	0.92 (0.50, 1.33)	0.015
3-RIR	0.58 (0.10, 1.06)	0.139
FAIL	2.32 (1.58, 3.05)	<0.001
**Female Participants**		
1-RIR	0.69 (0.35, 1.03)	0.076
3-RIR	0.11 (-0.41, 0.64)	0.766
FAIL	1.24 (0.71, 1.76)	0.003

S4. Repetition Loss from First to Final Set

4.1 Linear Mixed Effects Model (Protocol x Sex)

Effect	DF	*F*-Value	*P*-Value
Protocol	2	64.96	<0.001
Sex	1	0.76	0.382
Protocol x Sex	2	0.26	0.775

4.1.1 Tukey's Pairwise Comparisons

Protocol	ES (CI)	*P*-Value
1-RIR vs. 3-RIR	1.26 (0.56, 1.97)	<0.001
1-RIR vs. FAIL	-1.31 (-1.84, -0.78)	<0.001
3-RIR vs. FAIL	2.49 (1.67, 3.30)	<0.001

4.1.2 Descriptive Statistics (% Change in Repetitions from First to Final Set)

Protocol	Mean
1-RIR	-40%
3-RIR	-27%
FAIL	-54%
**Protocol x Sex**	
1-RIR *(Male)*	-44%
1-RIR *(Female)*	-37%
3-RIR *(Male)*	-30%
3-RIR *(Female)*	-25%
FAIL *(Male)*	-59%
FAIL *(Female)*	-51%

4.2 Within-Protocol Statistical Differences from First Set to Final Set

**Protocol**	**ES (CI)**	***P*-Value**
**All Participants**		
1-RIR	1.97 (1.50, 2.44)	<0.001
3-RIR	1.11 (0.73, 1.50)	<0.001
FAIL	2.64 (2.07, 3.22)	<0.001
**Male Participants**		
1-RIR	2.55 (1.59, 3.51)	<0.001
3-RIR	1.51 (0.63, 2.39)	<0.001
FAIL	3.72 (2.24, 5.20)	<0.001
**Female Participants**		
1-RIR	2.12 (1.28, 2.97)	<0.001
3-RIR	1.11 (0.54, 1.69)	0.008
FAIL	3.01 (1.93, 4.01)	<0.001

4.3 Repetitions Performed Per Set (Data Shown are Expressed as Mean ± *SD*)

Set	1	2	3	4	5	6
**All Participants**						
1-RIR	13 ± 3	11 ± 2	9 ± 2	9 ± 2	8 ± 2	8 ± 2
3-RIR	10 ± 3	9 ± 2	9 ± 2	8 ± 2	7 ± 2	7 ± 2
FAIL	14 ± 3	11 ± 3	9 ± 3	8 ± 3	7 ± 2	6 ± 2
**Male Participants**						
1-RIR	11 ± 2	9 ± 2	8 ± 2	7 ± 1	7 ± 1	6 ± 1
3-RIR	9 ± 2	8 ± 1	8 ± 2	7 ± 2	6 ± 1	6 ± 2
FAIL	12 ± 2	9 ± 2	7 ± 1	6 ± 2	5 ± 1	5 ± 1
**Female Participants**						
1-RIR	14 ± 2	12 ± 2	11 ± 2	10 ± 2	9 ± 2	9 ± 2
3-RIR	12 ± 3	11 ± 2	10 ± 2	10 ± 2	9 ± 2	9 ± 2
FAIL	16 ± 3	12 ± 3	11 ± 3	9 ± 3	8 ± 2	8 ± 2

S5. Perceived Discomfort

5.1 Friedman Test (Protocol)

**Effect**	**DF**	***Chi-Squared***	***P*-Value**
Protocol	2	30.98	<0.001

5.1.1 Tukey's Pairwise Comparisons

**Protocol**	**ES (CI)**	***P*-Value**
1-RIR vs. 3-RIR	0.62 (0.26, 0.99)	0.005
1-RIR vs. FAIL	0.65 (0.36, 0.94)	0.001
3-RIR vs. FAIL	1.50 (1.07, 1.93)	<0.001

5.1.2 Descriptive Statistics (Rating of Perceived Discomfort)

**Protocol**	**Mean**	***SD***
1-RIR	3.76	1.75
3-RIR	2.74	1.17
FAIL	4.88	1.48

S6. Perceived Exertion

6.1 Friedman Test (Protocol)

**Effect**	**DF**	***Chi-Squared***	***P*-Value**
Protocol	2	35.89	<0.001

6.1.1 Tukey's Pairwise Comparisons

**Protocol**	**ES (CI)**	***P*-Value**
1-RIR vs. 3-RIR	1.14 (0.66, 1.63)	<0.001
1-RIR vs. FAIL	0.95 (0.37, 1.53)	0.003
3-RIR vs. FAIL	1.85 (1.12, 2.57)	<0.001

6.1.2 Descriptive Statistics (Rating of Perceived Exertion)

**Protocol**	**Mean**	***SD***
1-RIR	4.33	1.40
3-RIR	2.88	0.74
FAIL	6.04	1.99

S7. General Feelings

7.1 Friedman Test (Protocol)

**Effect**	**DF**	***Chi-Squared***	***P*-Value**
Protocol	2	17.13	<0.001

7.1.1 Tukey's Pairwise Comparisons

**Protocol**	**ES (CI)**	***P*-Value**
1-RIR vs. 3-RIR	0.56 (0.12, 1.00)	0.025
1-RIR vs. FAIL	0.63 (0.08, 1.17)	0.071
3-RIR vs. FAIL	1.19 (0.58, 1.81)	0.001

7.1.2 Descriptive Statistics (Feeling Scale)

**Protocol**	**Mean**	***SD***
1-RIR	2.42	1.67
3-RIR	3.29	1.27
FAIL	1.25	1.92

S8. Muscle Soreness

8.1 Friedman Test (Protocol)

**Effect**	**DF**	***Chi-Squared***	***P*-Value**
Protocol *(24-hrs)*	2	18.40	<0.001
Protocol *(48-hrs)*	2	14.08	0.001

8.1.1 Tukey's Pairwise Comparisons

**Protocol**	**ES (CI)**	***P*-Value**
24-hrs		
1-RIR vs. 3-RIR	0.37 (-0.20, 0.94)	0.558
1-RIR vs. FAIL	0.79 (0.19, 1.39)	0.023
3-RIR vs. FAIL	1.16 (0.64, 1.68)	<0.001
48-hrs		
1-RIR vs. 3-RIR	0.40 (-0.06, 0.87)	0.316
1-RIR vs. FAIL	0.42 (-0.17, 1.00)	0.417
3-RIR vs. FAIL	0.90 (0.31, 1.50)	0.004

8.1.2 Descriptive Statistics (Rating of Muscle Soreness)

**Protocol**	**Mean**	***SD***
24-hrs		
1-RIR	2.29	1.71
3-RIR	1.67	1.52
FAIL	3.79	1.96
48-hrs		
1-RIR	1.5	2
3-RIR	0.79	1.14
FAIL	2.38	2.06

S9. Perceived Recovery

9.1 Friedman Test (Protocol)

**Effect**	**DF**	***Chi-Squared***	***P*-Value**
Protocol *(24-hrs)*	2	21.30	<0.001
Protocol *(48-hrs)*	2	12.83	0.002

9.1.1 Tukey's Pairwise Comparisons

**Protocol**	**ES (CI)**	***P*-Value**
24-hrs		
1-RIR vs. 3-RIR	0.75 (0.21, 1.29)	0.014
1-RIR vs. FAIL	0.52 (-0.05, 1.08)	0.204
3-RIR vs. FAIL	1.55 (0.83, 2.27)	0.001
48-hrs		
1-RIR vs. 3-RIR	0.66 (0.25, 1.06)	0.008
1-RIR vs. FAIL	0.24 (-0.33, 0.81)	0.659
3-RIR vs. FAIL	1.02 (0.45, 1.58)	0.003

9.1.2 Descriptive Statistics (Rating of Perceived Recovery Status)

**Protocol**	**Mean**	***SD***
24-hrs		
1-RIR	3.83	2.20
3-RIR	5.42	1.84
FAIL	2.88	1.23
48-hrs		
1-RIR	5.83	2.28
3-RIR	7.21	1.47
FAIL	5.29	2.07
